# The immunology of other mycobacteria: *M. ulcerans*, *M. leprae*

**DOI:** 10.1007/s00281-020-00790-4

**Published:** 2020-02-25

**Authors:** Katharina Röltgen, Gerd Pluschke, John Stewart Spencer, Patrick Joseph Brennan, Charlotte Avanzi

**Affiliations:** 1 Department of Pathology, Stanford School of Medicine, Stanford University, Stanford, CA USA; 2 Medical Parasitology and Infection Biology, Swiss Tropical and Public Health Institute, Basel, Switzerland; 3 University of Basel, Basel, Switzerland; 4 Mycobacteria Research Laboratories, Department of Microbiology, Immunology and Pathology, Colorado State University, Fort Collins, CO USA

**Keywords:** Buruli ulcer, Leprosy, Immune evasion, Polarization of immune responses, Immunopathology, Diagnosis, Vaccine design

## Abstract

Mycobacterial pathogens can be categorized into three broad groups: *Mycobacterium tuberculosis* complex causing tuberculosis, *M. leprae* and *M. lepromatosis* causing leprosy, and atypical mycobacteria, or non-tuberculous mycobacteria (NTM), responsible for a wide range of diseases. Among the NTMs, *M. ulcerans* is responsible for the neglected tropical skin disease Buruli ulcer (BU). Most pathogenic mycobacteria, including *M. leprae*, evade effector mechanisms of the humoral immune system by hiding and replicating inside host cells and are furthermore excellent modulators of host immune responses. In contrast, *M. ulcerans* replicates predominantly extracellularly, sheltered from host immune responses through the cytotoxic and immunosuppressive effects of mycolactone, a macrolide produced by the bacteria. In the year 2018, 208,613 new cases of leprosy and 2713 new cases of BU were reported to WHO, figures which are notoriously skewed by vast underreporting of these diseases.

## Introduction

Both *Mycobacterium ulcerans* and the leprosy bacillus have a low optimal growth temperature of 30–33 °C, which is considered a major factor for the skin tropism and the limited systemic dissemination of the infections they cause. Both pathogens cause chronic infections with serious skin manifestations but have developed two very distinct survival strategies in the human host. In advanced BU lesions, clusters of extracellular *M. ulcerans* reside in necrotic tissue areas largely devoid of infiltrating leukocytes and thus mostly inaccessible to the immune system [1]. Immune evasion of *M. ulcerans* is facilitated by mycolactone and in addition by the loss of immunodominant antigens during the course of reductive evolution [2, 3]. Also *M. leprae* has adapted to mammalian host cells via rigorous genome reduction and loss of gene function, but in contrast to *M. ulcerans*, *M. leprae* has evolved to become an obligate intracellular microorganism, able to invade, and replicate in phagocytic cells and Schwann cells [4]. Polarization of the immune response toward either T_H_1-type cytokine profiles restricting growth of the bacilli or T_H_2-type responses resulting in progressive infection has been identified as one of the key determinants for the outcome of an exposure to *M. leprae* in mouse infection models. However, other T lymphocyte subsets identified in recent years in humans may cause effects that go beyond this classical T_H_1/T_H_2 paradigm [5]. Considering evidence of an intracellular stage of *M. ulcerans* early after infection [6], cell-mediated immunity may also be important for the containment of BU.

A good understanding of the mechanisms of immune evasion is of key importance for the development of vaccines, rapid diagnostic tests (RDTs), and efficient treatment regimens. Both leprosy and BU patients develop early skin symptoms, often overlooked by patients and misdiagnosed by untrained clinicians [7, 8]. Lack of point of care diagnostic tests often hinders diagnosis in early disease stages, which is crucial to prevent the disabling and stigmatizing long-term effects of the diseases. Specific antibiotic treatment is available for both leprosy and BU and is—particularly in early disease stages—highly effective [9, 10]. Patient compliance with the required months-long regimens, which are known to cause severe side effects, and a constant supply of these drugs to the often remote, endemic areas are major challenges. Prevention of BU and leprosy with an efficient vaccine is a long-desired solution to protect individuals from severe pathology and to potentially also interrupt transmission. In this review, we summarize current knowledge on the immunology and immunopathology of BU and leprosy and discuss implications for the design of effective vaccines and point of care diagnostics.

### Buruli ulcer

#### Emergence of *M. ulcerans* and pathogenesis of BU

BU is a chronic, necrotizing skin disease caused by infection with *M. ulcerans* [11]*.* Infection foci are closely associated with slow-flowing and stagnant water bodies, but transmission pathways of *M. ulcerans* are not fully understood. Whole-genome sequencing analyses indicate that *M. ulcerans* has evolved from the environmental bacterium *M. marinum* by acquisition of a virulence plasmid (pMUM) [2, 12], comprising genes that code for polyketide synthases and polyketide-modifying enzymes required for the biosynthesis of macrolide toxins designated mycolactones [13]. Subsequent reductive evolution and pseudogene formation, most likely reflecting an adaptation to a more stable ecological niche, were mainly mediated by the proliferation of acquired insertion sequence (IS) elements, such as IS*2404*, present in the genome of *M. ulcerans* in high copy numbers [2]. In the course of its evolution, *M. ulcerans* has diverged into several niche-adapted lineages (ecovars). The natural host range of these ecovars together is broad, including humans, other mammals, and ectotherms such as fish and frogs [14]. Two principal lineages have been identified among isolates from human lesions. Strains of the highly clonal classical lineage are isolated from BU cases in Africa, Papua New Guinea, and Australia, where disease hot spots with high local prevalence are found. In contrast, the more scattered BU cases identified occasionally in Asia and the Americas are caused by strains of the ancestral lineage [15]. All mycolactone-producing mycobacteria (MPM) seem to carry basically the same virulence plasmid pMUM, but due to minor sequence modifications in the plasmid, they produce different molecular variants of mycolactone with different biological activities [16–18]. In addition, ecovars differ substantially in the patterns of genomic deletions and point mutations in the main chromosome accumulated during evolution from a common ancestor that had acquired pMUM. Both the production of a particular variant of mycolactone and the pattern of genetic changes in the chromosome seem to influence virulence and host range of the different ecovars.

In BU patients who acquired an *M. ulcerans* infection during a defined short stay in an endemic area of Victoria (Australia), the mean incubation period was 4.5 months with a wide variation from 32 to 264 days [19]. BU usually starts with the formation of a single, painless, subcutaneous nodule or papule, most commonly located on the extremities. In some cases, other non-ulcerative forms—plaques or edema—develop. The dermis and epidermis overlying these closed lesions may eventually slough off, leading to the development of ulcers with undermined edges and a necrotic slough at the base. Coagulation necrosis of the deep dermis and subcutaneous fat tissue, destruction of blood vessels, and interstitial edema are characteristics of advanced lesions [20]. Clusters of acid-fast bacilli (AFBs) are primarily located in deep layers of a central amorphous coagulum [21]. The pathology induced by ancestral and classical lineage strains may be equally severe.

#### The central role of mycolactone in the pathogenesis of BU

**Fig. 1:**
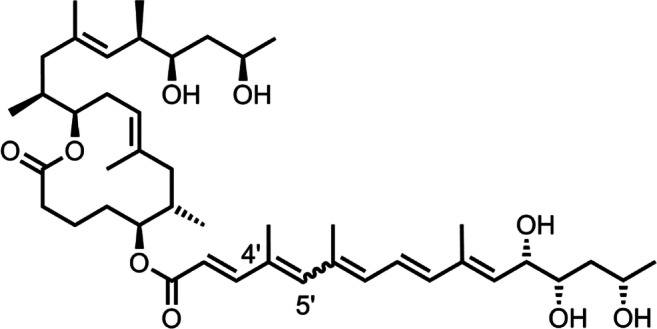
Structure of mycolactone A/B

Mycolactones have cytotoxic, analgesic, and immunosuppressive properties and play a key role in the pathology of BU [22]. They consist of a 12-membered lactone ring, a C5-O-linked polyunsaturated acyl side chain, and a C-linked upper side chain comprising C12–C20 [13] (Fig. 1). Whereas the structures of the core lactone ring and the upper side chain are conserved, mycolactones isolated from the different *M. ulcerans* ecovars vary in length, number of double bonds, and number/localization of hydroxyl groups of the lower side chain. Individual mycolactones are a mixture of two stereoisomers that interconvert. The mycolactone predominantly produced by African classical lineage *M. ulcerans* disease isolates is therefore designated mycolactone A/B.

Injection of mycolactone into the skin of experimental animals produces lesions similar to those of human BU [23], demonstrating the key role of the toxin in the pathogenesis of *M. ulcerans* disease. Furthermore, infection of mice with *M. ulcerans* wild-type strains, but not with mycolactone-negative mutants, causes the chronic, necrotizing lesions characteristic for BU [24, 25]. Mycolactones seem to diffuse passively through the host cell membranes and may alter the dynamic properties of the plasma membrane. They promote Bim-dependent apoptosis of mammalian cells through the Akt-FoxO3 axis. Accordingly, *M. ulcerans-*infected Bim knockout mice are able to contain mycobacterial multiplication and do not develop necrotic BU lesions [26]. Sec61 blockade appears to play a key role in the initiation of mycolactone-induced cell death by activation of an integrated stress response [22]. In vitro, exposure of different types of mammalian cells to low nanomolar concentrations of mycolactone A/B leads within 2 to 3 days to cell detachment and death [27].

Mycolactones are produced locally by clusters of *M. ulcerans* located in the infection foci in the skin but have also been found in peripheral blood cells, spleen, liver, and kidneys of experimentally infected mice [28]. Non-toxic concentrations of mycolactone in the periphery may have suppressive effects for the systemic immune system. Locally, macrophages and lymphocytes accumulate at the margin of the necrotic center of BU lesions, but cells infiltrating the necrotic core seem to be killed by a cloud of mycolactone surrounding the *M. ulcerans* clusters.

#### Other virulence factors of *M. ulcerans*

The acquisition of the virulence plasmid pMUM by *M. ulcerans* constitutes an evolutionary bottleneck event, most likely creating the basis for the development of a new, highly clonal species with increased virulence. There is no doubt that the cytotoxic and immunosuppressive properties of mycolactone are pivotal for the pathogenesis of BU, but other factors may contribute to the pathologic process. Genome comparison between *M. ulcerans* and *M. marinum* revealed more than 97% overall nucleotide identity between the two pathogens and only limited *M. ulcerans* species-specific content, except for pMUM [2]. Considering the massive loss of DNA and extensive pseudogene accumulation in the genome of *M. ulcerans* compared with that of *M. marinum*, immune evasion appears to be a pathogenic strategy of *M. ulcerans*. This is highlighted by the loss of expression of strong B and T cell immunogens, such as the highly immunogenic 6-kDa early secreted antigenic target protein (ESAT-6), the 10-kDa culture filtrate protein (CFP-10), and the heat shock protein X (HspX) [3]. In *M. tuberculosis* infections, the ESAT-6 protein secretion system (ESX-1) is implicated in the initiation of granuloma formation, phagosome maturation, and bacterial dissemination between macrophages, and is thus considered a major virulence determinant [29, 30]. Independent deletions or loss of gene function mutations in genes of the ESX-1 locus in *M. ulcerans* strains indicate a selective advantage conferred by the loss of these major immunogens, and may also contribute to the predominantly extracellular lifestyle of *M. ulcerans* [3]. Moreover, it has been shown that *M. ulcerans* is surrounded by an extracellular matrix, containing not only mycolactone but also a variety of proteins, carbohydrate structures, and lipids/lipoglycans, such as lipoarabinomannan, phosphatidylinositol mannosides, and phthiocerol diesters, known to be involved in the virulence of other mycobacterial species [31]. It is assumed that this layer shields the extracellularly replicating mycobacteria from effector mechanisms of the immune system. Also, phospholipase C activity associated with virulence in other pathogenic mycobacteria was reported in *M. ulcerans* [32]. Genetic studies on other potential virulence factors have been hampered by the extremely slow growth of the bacteria (colonies appear only after several weeks on solid media) and the lack of methods for systematic knockout of candidate genes.

#### Cell-mediated vs humoral immunity

Infection with *M. ulcerans* leads to progressive skin lesions, usually without distinct systemic symptoms. Knowledge on types of immune responses involved in interactions with *M. ulcerans* and potential mechanisms conferring protection from BU is still very limited. In histopathological analyses of tissue sections from advanced BU lesions, extracellular clumps of AFBs are found in extensive necrotic areas with an apparent lack of infiltrating leukocytes. Hence, *M. ulcerans* has long been defined as a primarily extracellular pathogen [20, 25]. The described histopathological hallmarks stand in stark contrast to the characteristic localized inflammatory response and granuloma formation seen in infections with facultative intracellular mycobacteria, such as *M. tuberculosis*, and have been associated with the cytotoxic and immunosuppressive activities of mycolactone [24, 25]. Considering the extracellular location of *M. ulcerans*, humoral immunity and, in particular, neutralizing antibodies against mycolactone could be a potent factor for protection. Surprisingly, a striking lack of *M. ulcerans*-specific antibody responses in the course of experimental infections with *M. ulcerans* was observed in the BU mouse model [33, 34]. Also, only one-third of all sera from BU patients analyzed in the framework of sero-epidemiological studies for BU contained antibodies against the immunodominant 18-kDa small heat shock protein (18 kDa shsp) of *M. ulcerans*, a fraction that was similar to that of healthy controls living in the same BU endemic area [35]. Lack of antibody responses may be attributed to the immunosuppressive effects of mycolactone. The concept that immune protection against infection with *M. ulcerans* is possible is supported not only by studies demonstrating *M. ulcerans-*specific humoral immune responses in individuals living in BU endemic areas without signs of active disease [35, 36] but also by reports on spontaneous healing of BU lesions [37, 38]. It remains to be investigated whether spontaneous healing is associated with the development of a specific immune response to *M. ulcerans* antigens.

As BU patients typically present late to health facilities, early BU lesions have not been well characterized by histopathology. A recent analysis of tissue sections from early lesions excised before antibiotic treatment from Australian BU patients has however revealed massive leukocyte infiltration surrounding the necrotic core of lesions [1]. An increasing body of evidence suggests that *M. ulcerans* may be transiently intracellular during the establishment of an infection [6, 39], and that cell-mediated immunity might thus play a key role in the containment of early infections. A strong argument for this view is the development of delayed-type hypersensitivity reactions in response to an intradermally injected extract of *M. ulcerans*, named Burulin [40], particularly in BU patients with healing lesions [36, 40], indicating an acquired T cell sensitization. Moreover, uptake of *M. ulcerans* by macrophages was demonstrated not only in vitro and in the BU mouse model [6] but also in tissue specimens of BU patients, where AFBs were detected inside phagocytes at the periphery of the infiltration belt surrounding the necrotic lesion core [1, 6].

It may be hypothesized that T_H_1 responses in early *M. ulcerans* infections can under certain circumstances be effective in preventing disease by activating macrophages to kill the phagocytosed bacilli. In contrast, a chronic infection may develop if early immune responses are not capable of eliminating the mycobacteria and a protective cloud of mycolactone eventually prevents infiltrating leukocytes from reaching the then extracellularly replicating bacilli, leading to the development of chronic lesions that may expand for many months or even years. To date, it is not known in what proportion of patients spontaneous healing occurs and which factors and processes allow the immune system to become rampant and induce a regressive state of the disease.

The outcome of an infection with *M. ulcerans* may depend on several factors, including dose of the initial inoculum, immunocompetence of the host, and prevailing cytokine environment polarizing the cellular immune system toward T_H_1 or T_H_2 responses. Highly conflicting results on the expression of specific cytokines in different stages of *M. ulcerans* infections have been published [41]. In general, *M. ulcerans* infection seems to impair the normal development of inflammatory and cellular immune responses and to suppress expression of most cytokines. The strong immunosuppressive activity of mycolactone is related to targeting the pore-forming subunit of the eukaryotic Sec61 translocon [22]. Sec61 is a heterotrimeric complex, which mediates the transport of secretory proteins into the ER, including cytokines and homing receptors of immune cells. Type I and type II transmembrane proteins seem to be generally susceptible to mycolactone-mediated Sec61 blockade, and this blockade also causes secondary proteomic alterations beyond Sec61 substrates. For example, the activation of multiple interferon (IFN)-responsive genes, including the nitric oxide synthase (iNOS) implicated in the control of mycobacterial infections, is impaired by inhibiting the production of both IFN-γ and its receptor [42]. That IFN-γ is an important factor in the early immune defense against *M. ulcerans* infection was demonstrated in the experimental BU mouse model, comparing wild-type and IFN-γ knockout mice. In IFN-γ–deficient mice, a significantly higher bacterial burden was detected 5 weeks after infection; disease progression was accelerated and led to more extensive tissue necrosis and edema [34]. Another proposed cellular effect of mycolactone relevant for immunosuppression is the activation of the Wiskott-Aldrich syndrome protein (WASP) family of actin-nucleating factors, affecting assembly of actin and cell-matrix adhesion [43].

#### Diagnosis in reference laboratories and at the point of care

Due to the wide spectrum of BU disease manifestations and the high prevalence of skin conditions with similar presentations (particularly in tropical BU endemic regions), the clinical diagnosis of BU is not always straightforward. PCR tests targeting the IS*2404* element of *M. ulcerans* are currently the gold standard for laboratory diagnosis of BU [7]. Swab samples from ulcers and fine needle aspirates from closed lesions are used for laboratory testing. A main drawback in implementing PCR-based assays is the indispensability of a good laboratory infrastructure (three-room principle to avoid contaminations), well-trained laboratory personnel, and rigid adherence to quality control measures. In the BU endemic African countries, current in-country PCR reconfirmation is restricted to only a few reference laboratories. Transport of clinical specimens from remote areas to these centers is associated with major logistical challenges, and treatment is often initiated based only on clinical signs and symptoms. Furthermore, an external quality assessment program for PCR diagnosis of BU has revealed that many reference centers have contamination problems, leading to false positive results [44].

Microscopy of Ziehl-Neelsen (ZN)–stained smears to detect AFBs is at present the only laboratory test for BU available at peripheral treatment facilities. As it has low sensitivity and specificity, there is urgent need for a simple point of care diagnostic test meeting the ASSURED (Affordable, Sensitive, Specific, User-friendly, Robust and rapid, Equipment free, and Deliverable) criteria outlined by the World Health Organization (WHO). Serological cross-reactivity of mycobacterial species, including *M. bovis* Bacillus Calmette-Guérin (BCG), with *M. ulcerans* has complicated the design of a species-specific serological test for BU. Furthermore, analyses of antibody responses against the well-characterized immunodominant 18 kDa shsp have revealed that also a substantial proportion of healthy individuals living in BU endemic areas develop *M. ulcerans-*specific immune responses [35]. Serological tests therefore seem to be suitable only for sero-epidemiological studies. Development of a BU-specific T cell–based assay similar to the IFN-γ release assays for tuberculosis failed because expression of immunodominant antigens, including ESAT*-*6 and CFP-10, was lost by *M. ulcerans* during the course of its reductive evolution [3]. In any case, serological and T cell–based assays for BU may not be practical due to mycolactone-mediated immunosuppression in patients with active disease. Therefore, ongoing efforts for RDT development for BU are focused on antigen detection assays targeting either *M. ulcerans-*specific proteins or mycolactone.

#### Treatment, immune reconstitution, and paradoxical reactions

Before the document “Provisional guidance on the role of specific antibiotics in the management of Mycobacterium ulcerans disease” was issued by WHO in 2004 [45],^1^ recommending a combination therapy with oral rifampicin (RIF, 10 mg/kg) and intramuscular streptomycin (STR, 15 mg/kg) for 8 weeks, surgery with wide excision margins had been the mainstay of BU treatment. In BU endemic regions of Africa, such interventions overburdened the available limited surgical infrastructure. Wide excisions were not well accepted by patients, but were necessary, as treatment failure rates of up to 47% were reported from hospitals, where more limited excisions were made [46]. In a formal clinical trial in patients with BU lesions with a diameter of < 10 cm, comparing the RIF/STR treatment with RIF plus STR for 4 weeks followed by RIF plus oral clarithromycin (CLA, 7.5 mg/kg) for 4 weeks, 96% of patients in the RIF/STR group and 91% in the RIF/STR-CLA group had healed lesions after 1 year. No recurrence was observed [9]. Low recurrence rates after antibiotic treatment have also been seen in several observational studies, although lesions may still be culture-positive after completion of the 8-week antibiotic treatment. Mycobacterial debris remaining in the lesions after chemotherapy may stimulate protective immune responses. In contrast, removal of most of the mycobacterial material by surgical excision may prevent such a “natural vaccination” effect. However, no formal long-term study assessing the relative reinfection risk of cured BU patients has been conducted so far.

BU lesions frequently show a transient worsening during antimycobacterial therapy. The size of the lesions may increase, new lesions may appear, and closed lesions may ulcerate before healing sets in [47]. These effects are usually related to prior massive destruction of subcutaneous tissue, not obvious before the upper layer of the skin sloughs off. Furthermore, an immune reconstitution inflammatory-like syndrome may cause paradoxical reactions. In tuberculosis, these are particularly common in human immunodeficiency virus (HIV)-positive individuals under antiretroviral therapy [48]. As mycolactone levels are declining in BU lesions under antimicrobial chemotherapy, local immunosuppression wanes and infiltrating leukocytes are no longer killed. As a consequence, vigorous local immune responses set in, and atopic lymphoid tissue develops at the site of the treated BU lesion [49]. In most patients, wound healing commences irrespective of the massive local leukocyte infiltration and stimulation, which can thus be regarded as a sign of the success of antimycobacterial treatment. In patients showing severe paradoxical reactions, the use of corticosteroids and/or adjunct surgical procedures has been proposed to prevent further tissue loss [50, 51].

#### Host factors and susceptibility to BU

BU in Africa affects mainly children between 4 and 15 years of age [52–54], whereas in endemic communities in Southeastern Australia, a high prevalence of BU is seen in the elderly [55]. There is an overall balanced gender distribution among BU cases, although several studies reported that, in children below 15 years of age, the disease is more common in boys than in girls, whereas in adults, infections are more frequent in females than in males [53, 54, 56]. Increased exposure of certain parts of a population to environments contaminated with *M. ulcerans* has been suggested as the main reason for this distinct age and gender distribution, but varying susceptibility should also be considered.

In contrast to children, adults may develop a certain degree of immunity after being exposed to *M. ulcerans*, whereas the elevated risk of developing BU in the elderly [52] may be related to the natural deterioration of the immune system with age. This hypothesis is corroborated by results from sero-epidemiological studies which indicate that toddlers are considerably less exposed to *M. ulcerans* than older children and adults of all age groups [35]. Exposure to *M. ulcerans* thus appears to increase at an age when children move further away from their homes and have more intense environmental contacts, including exposure to water bodies at the periphery of their home villages. Apart from differences in environmental contact patterns, reasons for the observed gender differences in susceptibility may in part be found in sex-specific genes or postpubertal hormones influencing several pathways of innate and acquired immune responses [57] or potentially interfering with effector mechanisms of mycolactone.

Host characteristics influencing the risk of developing BU disease are not well understood, as it is difficult to recruit sufficient numbers of participants for case-control studies within a reasonable timeframe. Therefore, there is paucity of data on further predisposing factors, such as infection with other pathogens prevalent in BU endemic areas, nutritional status, and host genetic factors. Assuming that cell-mediated immunity plays a key role in the early defense against *M. ulcerans* infection, and that the depletion of CD4 T cells in the acquired immunodeficiency syndrome (AIDS) caused by HIV weakens T_H_1 responses, BU-HIV coinfection has gained particular attention. In case-control studies and reports on BU case series, it was shown that the prevalence of HIV in BU patients is significantly higher than that in the local control population [58–60]. Moreover, BU-HIV coinfected patients appear to have more severe skin lesions and more often multifocal lesions than HIV-negative BU patients [59, 60]. Potential associations of BU with other infections are yet to be investigated. It had been hypothesized that helminth infections which drive the immune system toward a T_H_2 response, may enhance susceptibility to *M. ulcerans* infection. However, a case-control study assessing the potential association of BU with helminth infections found no difference in the prevalence and burden of schistosomes in BU patients and controls [61].

Differences in host genetics may also play a role in susceptibility to and outcome of *M. ulcerans* infections. Polymorphisms in candidate genes that have been analyzed for a potential effect on BU were primarily selected based on their documented association with susceptibility to other mycobacteria. It may nevertheless be of significance that several single nucleotide polymorphisms (SNPs) associated with susceptibility to BU are implicated in resistance to intracellular pathogens, further stressing the likely role of macrophages for the early containment of *M. ulcerans* infections. Associations of susceptibility to *M. ulcerans* disease have been found for SNPs, reducing the promoter activity of the inducible nitric oxide synthase gene *iNOS* and of the *IFN-γ* gene and SNPs in the natural resistance-associated macrophage protein gene *SLC11A1* (*NRAMP1*), all implicated in the regulation of macrophage activation [62, 63]. Relevant polymorphisms have also been detected in the autophagy-related E3 ubiquitin protein ligase gene PRKN (formerly *PARK2*) implicated in ubiquitin-mediated autophagy of mycobacteria [64, 65]. In contrast to candidate gene studies, genome-wide association studies based on comparison of the frequency of large numbers of SNPs across the genome of patient cohorts and controls have the power to discover new factors specifically associated with susceptibility to the disease of interest. It is however doubtful whether this approach can be applied to BU, considering the difficulty to recruit the very high numbers of participants required to reach statistical significance in this type of comprehensive studies.

#### Limited BCG vaccine efficacy and prospects for alternative vaccine design

No vaccine is currently available to effectively protect affected populations from BU, and reports on a species cross-reactive protective effect of the TB vaccine BCG have been inconsistent. Whereas case-control studies conducted in several West and Central African countries to specifically assess BCG vaccine efficacy against BU found no evidence of a protective effect [66, 67], having a BCG scar or vaccination record was correlated with protection against *M. ulcerans*-associated osteomyelitis in a case series in Benin [68, 69]. Limited numbers of study participants and retrospective determination of vaccination status by BCG scar readings are potential confounders in these studies. In a randomized BCG vaccination trial for BU conducted in the 1970s in Uganda, BCG was found to offer short-lived protection of 63% in the first year after vaccination and close to no protection in subsequent years [70], reconfirming results of a previous smaller trial [71]. Apart from the perspective of waning immunity over time, the very abrupt decline in protection may be explained by a mere delay in the onset of symptoms in those with an already established *M. ulcerans* infection. This is indicated in the mouse model of experimental *M. ulcerans* infection, where BCG induces an immune response capable of transiently containing *M. ulcerans* proliferation but ultimately failing at preventing the development of typical BU pathology [72]. In this model, protection is not prolonged by BCG booster vaccination [73].

Rational vaccine design for BU is complicated by limited knowledge on correlates of protection. Upon infection with *M. ulcerans*, vaccine-induced immune responses should ideally target the postulated early intracellular phase of *M. ulcerans* by cell-mediated responses. That way the initial inoculum may be eliminated before extracellular bacterial clusters can form. In this advanced stage of the infection, mycobacteria are accessible for antibodies but are protected from infiltrating leukocytes by the immunosuppressive and cytotoxic actions of mycolactone [1].

Prompted by the observed transient protective effect of BCG, the potential of genetically engineered BCG as a vehicle for BU vaccine development has been explored in the BU mouse model. Recombinant BCG-based vaccine candidates overexpressing immunodominant *M. ulcerans* proteins such as Ag85A or Ag85B-EsxH were able to initiate antimycobacterial T_H_1-type responses and to significantly, but only transiently, enhance protection from experimental *M. ulcerans* infection compared with the standard BCG vaccine [74, 75]. Other mycobacterial species with greater antigenic homology to *M. ulcerans*, such as *M. marinum*, may provide a richer source of protection-relevant immunogens. Indeed, transient protection against *M. ulcerans* infection after immunization of mice with *M. marinum* was shown to be superior to that conferred by BCG [76]. Mycolactone-negative mutants [72] or other attenuated *M. ulcerans* strains [77] may represent an alternative approach to live vaccine design, although initial results fell short of expectations [72]. Although capable of inducing antigen-specific T_H_1-type responses, DNA-based subunit vaccines encoding different immunogenic proteins, such as Hsp65, Ag85A, and polyketide synthase domains involved in mycolactone synthesis, had only limited efficacy in mice [78–80]. A combined DNA prime-protein boost protocol led to transient protection comparable with that provoked by the BCG vaccine [78]. Strong humoral immune responses induced by immunization of mice with two adjuvanted recombinant *M. ulcerans* proteins failed to provide protection against subsequent *M. ulcerans* infection, indicating that opsonization of the bacteria is not sufficient to prevent bacterial multiplication.

Taking the results obtained with live and subunit vaccine candidates together, it appears that targeting the immunosuppression caused by mycolactone itself may be the most promising approach for the development of a prophylactic and/or therapeutic BU vaccine. Until recently, all attempts to raise antibodies against this poorly immunogenic, cytotoxic macrolide had failed. Immunization with a carrier protein conjugate of a non-toxic synthetic truncated mycolactone derivative has enabled for the first time the generation of anti-mycolactone antibody responses in mice. Mycolactone-specific immune sera and monoclonal antibodies showed toxin-neutralizing activity, preventing mammalian cell apoptosis in an in vitro assay [81]. It remains to be seen whether vaccine-induced mycolactone-specific humoral immune responses on their own are protective in vivo*.* Alternatively, prevention of mycolactone-mediated immunosuppression may have to be combined with the induction of protective immune responses against *M. ulcerans* protein antigens.

### Leprosy

#### Pathogenesis of leprosy

Leprosy is a multiform disease mainly caused by *Mycobacterium leprae* and to a lesser extent by *M. lepromatosis*^2^ [82]. The disease affects many tissues, but one of the first signs that lead leprosy patients to seek medical consultation are dermatological changes, including various numbers and types of skin lesions with central loss of pigmentation and loss of sensitivity [8]. The second hallmark of leprosy is peripheral neuropathy, leading to severe and irreversible deformities and disabilities in the absence of prompt treatment [8]. The incubation time of the disease ranges from a few months to > 20 years [4], and the appearance of symptoms is usually slow and insidious, which can negate the opportunity for early treatment [4]. If untreated, leprosy lesions and tissue damage can lead to multi-organ failure, directly or indirectly due to bacterial infection and peripheral neuropathy [8]. Leprosy can present as an early and transitory form characterized by one or several hypopigmented macules and evolve either to spontaneous healing or progress toward a broad spectrum of clinical presentations for which severity differs significantly between patients [4]. Systemic infections are rare in leprosy, and their etiologies are unknown.

**Fig. 2:**
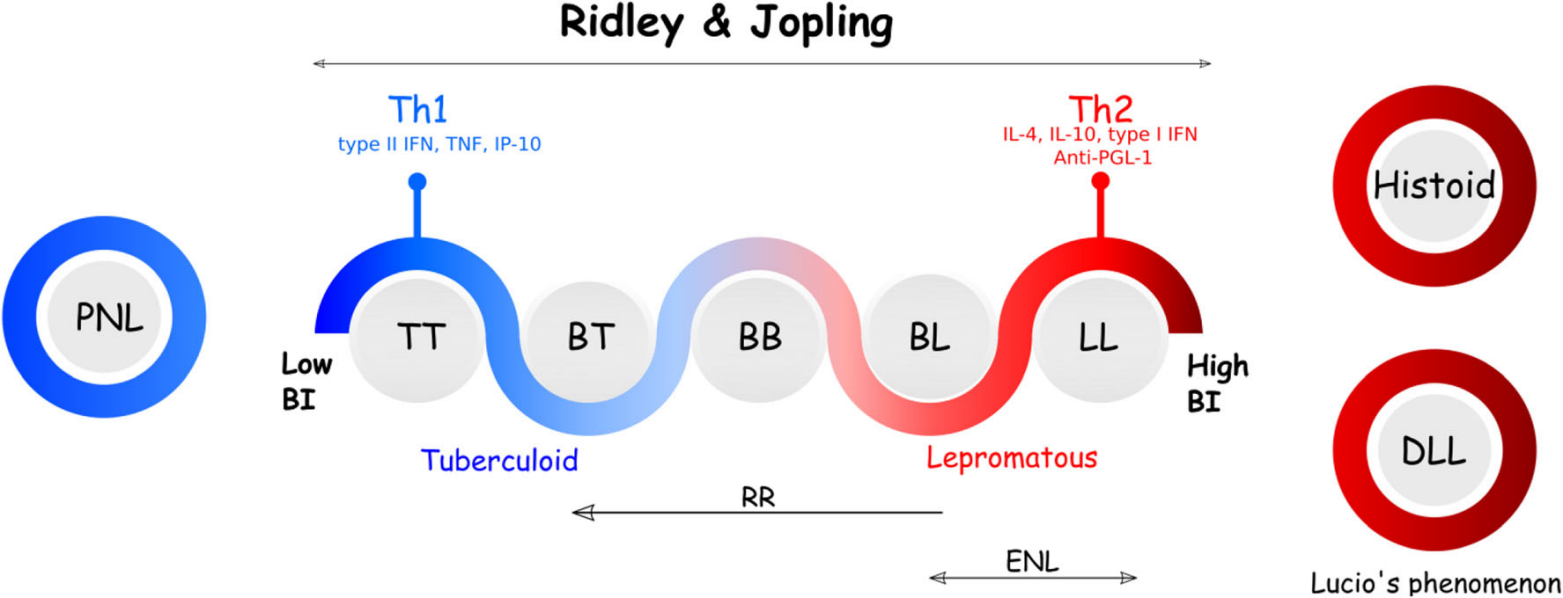
Ridley and Jopling classification and unusual forms of leprosy, associated with the degree of the bacillary load and the immune response. *PNL* pure neural leprosy, *DLL* diffuse lepromatous leprosy, *LL* lepromatous leprosy, *BL* borderline lepromatous leprosy, *BB* borderline borderline leprosy, *BT* borderline tuberculoid leprosy, *TT* tuberculoid leprosy. Adapted from [150]

When entering the human body, probably through the nose or the skin, the bacteria invade, survive, and multiply within phagocytic cells, keratinocytes, and Schwann cells through several pathways. These cells contain receptors sensing different bacterial components, and once activated, they generate inflammatory responses, secreting cytokines to activate other cells of the immune system and to activate T cells by presenting antigens to them [83]. The Ridley and Jopling classification (Fig. 2) is one of the most common protocols used by dermatologists to characterize clinical presentations of leprosy [8]. The classification consists of five categories based on the bacterial load as well as the clinical, histological, and immunological features in skin biopsies: polar tuberculoid leprosy (TT), with the highest cellular response (type II interferon IFN-γ, tumor necrosis factor alpha (TNFα), and interleukin (IL)-15) and a restricted growth of bacteria, followed by borderline tuberculoid (BT), borderline borderline (BB), borderline lepromatous (BL), and the polar lepromatous type (LL), characterized by an increased but inefficient humoral response (IL-4 and IL-10) leading to the survival of bacteria in the host [8]. Pure neural leprosy (PNL) is one of the challenging tuberculoid forms to diagnose because skin lesions are mostly absent and only nerve swelling and pain, detected by palpation, characterize the disease. The diffuse lepromatous leprosy (DLL) and the histoid leprosy forms are rare, but severe lepromatous leprosy forms are considered to be extremely infectious [8]. Based on the bacterial burden, leprosy patients are also classified as paucibacillary (PB) or multibacillary (MB) cases.

During the course of active disease and even after treatment, 30–50% of leprosy patients experience severe immunological complications called leprosy reactions. Reactions are unpredictable and initiated by an acute inflammatory episode inducing systemic, neuropathic, and cutaneous modification leading to a high level of morbidity without rapid treatment with steroid drugs [8, 84]. Reversal reaction (RR or type 1 reaction, T1R) is characterized by a rapid increase in the cellular T_H_1 response against *M. leprae.* It occurs in around 30% of cases, mainly in the borderline spectrum, resulting in an upgrading of the disease toward the tuberculoid side [85]. The inflammation is localized in skin lesions and/or nerves and characterized by local changes of IFN-γ and TNF-α secretion by the CD4+ lymphocytes and a decrease in IL-10 [85, 86]. Similar to RR, erythema nodosum leprosum (ENL, type 2 reaction, T2R) is associated with painful inflammation of skin lesions and nerves but extending over several years with recurrent or chronic episodes [87]. ENL occurs in 50% of lepromatous and 5–10% of borderline lepromatous cases [88]. A possible mechanism involves the release of inflammatory cytokines such as TNF-α in the lesions following neutrophil influx [87]. However, this influx is mainly observed in the 72 h following the onset of symptoms and almost absent when measured a few days later, suggesting that these cells play a major role in the evolution of ENL [89]. Anti-TNF-α drug therapies are being examined for the treatment of acute phases of ENL, but further work is needed to understand the physiopathology of ENL, especially the difference between acute, recurrent, and chronic forms, to find new targets for intervention and to improve treatment [87].

The third response, named Lucio’s phenomenon, is a rare but life-threatening reaction mainly associated with the diffuse lepromatous leprosy form and sometimes with lepromatous leprosy [8]. Lucio’s phenomenon clinically presents with extremely severe symptoms of skin lesions surrounded by erythema progressing to necrosis of the skin. Similar to ENL, deposits of immune complexes in dermal blood vessels seem to be associated with the reaction, but the exact immunopathogenesis remains unknown due to the paucity of cases and research studies [90].

Mechanisms by which the patient will develop one or the other form are currently unknown, but the type of host immune response is one of the main determinants (Fig. 2). However, immune responses are different when studied locally in skin lesions or in the blood, suggesting that besides immunological responses, other factors such as host genetics, environmental factors, bacterial genetics, and the route of infection, as well as the bacterial load, play a role in disease outcome [86, 91].

#### Immunological mechanisms of leprosy polarization

The initial interaction between *M. leprae* and the innate immune system is crucial to develop an efficient antimicrobial response, and the difference eventually leads to the generation of T_H_1- or T_H_2-like effector cells, characteristic of the polarized state of immunity in leprosy [92]. In the past decades, several mechanisms have been studied to link the different responses and to understand the host mechanisms that could lead to the leprosy spectrum. The main discoveries are discussed below.

#### Modulation of innate immunity

Unlike in *M. ulcerans*, there is no main virulence factor in *M. leprae*. However, most of the genes from the type VII ESX secretion and virulence system used to escape phagocytosis in mycobacteria are conserved including the membrane lysis substrate EsxA [82]. Native phenolic glycolipid I (PGL-I) is the most abundant and immunogenic *M. leprae* antigen. Several studies demonstrated that the trisaccharide structure is unique and specific for *M. leprae* PGL-I, which later became the basis for most of the serological tests developed over the past several decades [93]. However, PGL-I [94], prominent and important in the serodiagnosis of leprosy, has also been implicated directly in leprosy nerve damage. Rambukkana [95] had convincingly shown that PGL-I binds to the G-domain of laminin-2 on the surface of Schwann cells, which in turn binds to the dystroglycan complex in the cell membrane directly initiating nerve demyelination. However, Madigan had argued [96] that an initial interaction of PGL-I with macrophages was crucial to nerve damage. Clearly, the role of PGL-I in the molecular and immunological event leading to leprosy nerve damage within Schwann cells awaits precise definition.

Lipoarabinomannan (LAM) is the dominant component of the cell wall of *M. leprae*. In fact, the copious amounts of LAM recovered from extracts of *M. leprae* allowed the type of chemical analysis that established for the first time that the lipid entity is phosphatidylinositol mannoside to which is attached a mannan backbone with an attached single-branched arabinan chain. The termini of this arabinan chain are largely substituted by single or several mannoses important in the phagocytosis of LAM by macrophage and Schwann cells [97]. The LAM of *M. tuberculosis* is a major modulator in the immune response to tuberculosis [98]. However, little of the corresponding research has been conducted in the case of leprosy, and only two studies are reported in the literature [99, 100].

Once inside the host, the main cell wall antigens are recognized by the complement receptors and initiate phagocytosis [101]. In parallel, the pathogen-associated molecular patterns (PAMPs) are also detected by pattern recognition receptors (PPRs) such as toll-like receptors (TLRs) 1 and 2 and nucleotide oligomerization domain (NOD) proteins. TLR2/TLR1 heterodimers are activated by *M. leprae* triacylated lipopeptides in monocytes which mediate macrophage differentiation and the T_H_1 cytokine response by the secretion of the pro-inflammatory cytokines TNF-α, IL-12, and IL-15 [101]. IL-15 stimulates the vitamin D–dependent antimicrobial activity and increases the secretion of the antimicrobial peptide cathelicidin [101]. Interestingly, a recent study has shown that an efficient antimicrobial response is dependent on the initial amount of vitamin D substrate present in the macrophages, suggesting that host metabolism at the time of infection is essential to initiate an efficient immune response against *M. leprae* [102]. Another important PRR involved in innate immune activation is the cytosolic sensor NOD-2. The receptor recognizes *M. leprae* muramyl dipeptide (MDP) and preferentially induces dendritic cell differentiation through the secretion of the pro-inflammatory cytokine IL-32 [103]. In addition, NOD-2 activation initiates a leucine-rich repeat kinase 2 (LRRK2)-dependent pro-inflammatory response as well as autophagy. In parallel, it also recruits caspase I leading to activation of IL-1β, an essential mediator of inflammation, and probably activation of T_H_17 cells [104]. The pro-inflammatory cytokine pathways (TLRs, components of the vitamin D antimicrobial pathway and the NOD-2 signaling pathways) are reportedly upregulated in tuberculoid compared with lepromatous lesions and have been proposed to explain the different clinical outcomes of leprosy [92].

In contrast, the leukocyte immunoglobulin-like receptor (LIR) subfamily A member 2 (LIR-7/LILRB2) membrane protein was found to be significantly upregulated in lepromatous compared with tuberculoid lesions. Additional functional studies have revealed that activation of LIR-7 inhibited innate host defense by shifting monocyte production of T_H_1 cytokines (IL-12) to T_H_2 cytokines (IL-10) and suppressed TLR2/1-induced antimicrobial activity [105]. The described ligands of LIR-7 range from class I major histocompatibility antigens to intact pathogen or immune-modulatory proteins [106]. In leprosy infection, the ligand remains to be identified. Nevertheless, it seems likely that LIRs are additional key players in innate immune modulation but toward the lepromatous side of the spectrum.

Current data highlight the importance of interferon secretion and autophagy regulation in the polarization of leprosy. Indeed, type II IFN (IFN-γ) is preferentially expressed in tuberculoid lesions promoting an antimicrobial response, while the type I IFN (IFN-β) is mostly activated in lepromatous lesions and leads to survival and dissemination of the bacteria. IFN-β induces the secretion of IL-27–dependent IL-10 activation contributing to the suppression of the antimicrobial response by inhibition of IFN-γ secretion and increasing the phagocytic and bacterial uptake activity of macrophages as well as the survival of *M. leprae* in host cells [101]. Recent data suggest that *M. leprae* can directly regulate the type I IFN secretion via cytosolic detection of *M. leprae* nucleic acid through the stimulator of interferon genes (STING) pathway, promote mycobacterial survival, and inhibit autophagy through the interaction with the autophagosome [101, 107]. Autophagy is upregulated in tuberculoid lesions, inhibited in lepromatous cases, and restored in lepromatous lesions when patients experience an increased T_H_1 response during reversal reaction [107].

Nevertheless, the spark that triggers the regulation of these different pathways through activation or inhibition is still unknown. A tempting hypothesis is based on the importance of the transmission pathway and/or bacillary load. Tuberculoid forms may arise from an infection route, by which the immune system is able to efficiently contain the bacteria and/or from a low bacillary load. Bacterial sensing via PRRs may be efficient by triggering an adequate immune response; the low amount of bacteria may not allow DNA sensing by STING and IFN-γ is efficiently secreted; autophagy would be upregulated, and cell-mediated immunity would be the main actor in the onset of leprosy lesions. On the other hand, a high bacillary load evoked by a mechanism of transmission, in which the immune system would not be able to contain the infection, would lead to a massive release of bacterial DNA, activation of the anti-inflammatory response via type I IFN, inhibition of autophagy, and persistence of the bacteria characteristic of the lepromatous forms. However, there is currently no laboratory model available to test the impact of bacillary load on the immune response, and the exact route(s) of infection remain(s) to be elucidated.

#### Metabolic immunity

The *M. leprae* genome has undergone reductive evolution and massive pseudogenization during its intracellular adaptation. The consequence is a loss of essential genes of bacterial metabolism, which makes bacterial survival highly dependent upon host cell resources. Several studies have shown that *M. leprae* infection promotes modifications of host cell metabolism and results in conversion of the host immune response. One of the most important metabolic pathways in correlation with the spectrum of clinical forms is, among others, the regulation of lipid metabolism [101, 103]. Enhancement of lipid metabolism is mainly observed in lepromatous lesions with the appearance of foamy macrophages (Virchow cells) composed of host-oxidized phospholipids. Host-derived phospholipids can regulate the immune response by inhibition of the vitamin D receptor (VDR)/cathelicidin antimicrobial pathway and induction of IL-10. Accumulation of these lipids is directly modulated by *M. leprae* through different mechanisms, including modulation of the host lipid synthesis and enhancement of lipid droplet formation [101, 103]. Besides these regulatory effects, *M. leprae* survives and replicates inside the lipid droplets in the phagosome of immune cells. It is also hypothesized that *M. leprae* can use host-derived lipids as a source of nutrients, and virulence factors as fatty acids are the main in vivo source of carbon for the mycobacteria through β-oxidation and are used in the production of surface-exposed lipid virulence factors in *M. tuberculosis* [108]. Moreover, Moraes and colleagues also suggested that *M. leprae* could use lipids to cover and hide its surface antigens from innate immune receptors in the cytosol [103]. All three mechanisms have not yet been validated in *M. leprae* infection.

#### Additional players of adaptive immunity

Besides typical T_H_1/T_H_2 responses, the role of several other lymphocytes in the modulation of host immune response in leprosy has been studied. These include natural killer T cells (NKT), FOXP3+ regulatory T (Treg) cells, and T helper 17 cells (T_H_17) [109]. Interestingly, T_H_17 and Treg cells seem to be associated with leprosy outcome with different functions such as T_H_1 and T_H_2. T_H_17 cells produce pro-inflammatory cytokines (IL-17) and are stimulated by IL-1β and IL-23. Additionally, T_H_17 response interacts with the T_H_1 response in tuberculoid patients to control the proliferation and dissemination of *M. leprae* [110]. Tuberculoid lesions contain more Th17 cells than lepromatous forms. In contrast to Th17, Treg cells contain inflammation and downregulate the immune response by expressing immune checkpoint molecules such as expression of the cytotoxic T lymphocyte-associated protein 4 (CTL4) [111]. CTL4 antagonizes the activation of CD8+-T cells via competition with the CD28 ligand resulting in an anergic T cell population. Interestingly, there is also a higher expression of Treg and CTL4 in lepromatous lesions [110]. CD8+ T cells are more abundant in lepromatous than tuberculoid forms, and they failed to respond to *M. leprae* stimulation in vitro suggesting that CTL4 plays an important role in the polarization of leprosy [112]. However, most of the studies are contradictory, and additional studies with more patients and appropriate controls for each type of reaction are needed. The current work on these different lymphocyte subsets suggested a regulatory role in the panel of immune responses in leprosy. Research in the field is still at an early stage, and future work should bring additional understanding of the immunopathogenesis of leprosy.

#### Immunological mechanism of leprosy neuropathy

In addition to phagocytic cells, *M. leprae* has a predilection for Schwann cells, which are glial cells conferring protection to peripheral nerves. For a long time, it was assumed that once internalized in Schwann cells through the binding of the PGL-I and α2-laminin, *M. leprae* would persist and multiply, and in response, the Schwann cells would express HLA-class II, and activated CD4 T-cells would then initiate a chronic inflammatory reaction [4]. However, this hypothesis was challenged several times by the observations that the *M. leprae* bacillary load is low in the peripheral nerves of paucibacillary patients despite severe nerve injuries [4]. Nerve damage in tuberculoid patients was expected to be correlated with the cellular response, and a possible mechanism was described recently [96]. Using a zebrafish infection model, the authors suggested that bacteria could reach peripheral nerves through permissive monocytes and progress toward a neurotoxic macrophage population through the secretion of iNOS, causing mitochondrial swelling and myelin loss in axons [96]. In lepromatous cases, epithelioid cells are mostly absent, and nerve lesions are associated with uninhibited growth of *M. leprae* in Schwann cells.

In the nine-banded armadillo model, endothelial cells of the epineurial and endoneurial blood vessels are considered to be the main transport route of *M. leprae* to the nerves [113]. However, the full picture has still not been elucidated. Understanding this mechanism could also help identify new drug targets to prevent nerve damage.

### Other factors influencing immunopathogenesis of leprosy

#### Host factors and susceptibility to leprosy

Around 90% of individuals exposed to multibacillary patients will not develop any signs of leprosy [114]. From this observation, it is believed that most of the population is naturally resistant to leprosy. In contrast to tuberculosis, it is currently not known whether latent infection occurs in leprosy. However, if this were the case, we would expect to see more HIV coinfections, as is the case with tuberculosis. Published data suggested that both diseases progress independently and that the impact of HIV infection on the course of leprosy is limited [115]. Therefore, it is likely that host genetics plays an important role in innate resistance.

Host genetic markers in several pathways such as in innate immune recognition, type I IFN, autophagy, and lipid and energy metabolism have been associated with increased resistance or susceptibility to leprosy [114]. The most convincing evidence is available for polymorphic alleles in the regulatory regions of PRKN, coding for an E3-ubiquitin ligase designated Parkin, shown to increase susceptibility to leprosy in all populations [116]. Parkin is involved in ubiquitin-mediated autophagy and provides resistance to intracellular pathogens. Additionally, mutations in Parkin and the PRKN-interacting LRRK2 were associated with reversal reaction [117].

There are currently no universal genetic markers that can define leprosy-susceptible or leprosy-resistant individuals in all populations, and it is unlikely that host genetic factors are the only main determinant of leprosy polarization. However, investigation of these host genetic markers is important to understand the mechanism of pathogenicity of *M. leprae* as well as the immune response following *M. leprae* infection. Altogether, such data can help identify new targets to improve the management of leprosy disease and reactional states.

#### Bacterial genetic factors

In *M. tuberculosis*, multiple studies have shown that genetic diversity of the pathogen translates into differences in transmission of the disease, progression to active disease, virulence, and immunogenicity. For example, the Beijing-L2 sub-lineage strains have repeatedly been associated with increased virulence and faster acquisition of drug resistance [118]. In comparison, *M. africanum*, a bacterium belonging to the *M. tuberculosis* complex, shows a lower global prevalence, with the main distribution in West Africa, and is less virulent than other lineages [118].

In *M. leprae*, the current distribution of genotypes also points toward the existence of more successful genotypes (1D, 4N, 3I) compared with others (1B, 3L, 3M) rarely found in modern samples, suggesting a host–pathogen adaptation. However, the sampling size of *M. leprae* genotypes is shallow, and less than 200 genome sequences are currently available [119]. Additionally, our incapacity to grow the pathogen limited the investigation *in vitro* and *ex vivo*. The most notable advance on this aspect was published in 2018 by Sharma and colleagues [120]. They showed that the 4P genotype has a growth advantage when propagated in nine-banded armadillo compared with the genotype 3I, suggesting that genetic differences are responsible for the pathological variation. However, there is currently no evidence of an association between the host phenotype and the different bacterial genetic backgrounds.

A few highly mutated genes unrelated to drug resistance were reported recently by Benjak and colleagues [119]. The most polymorphic gene was a member of the PPE protein family, ML0411, encoding the serine-rich antigen. The high mutation rate in *ml0411* might reflect selection pressure from the human immune system. In addition, the fact that *ml0411* has no counterpart in other mycobacteria except for the other leprosy agent, *M. lepromatosis*, is further suggestive of a specific role in the pathogenesis of leprosy. Additional efforts should be deployed to functionally characterize these hypermutated genes and increase the number of *M. leprae* genomes sequenced in association with information on the clinical form of the disease.

#### Treatment and leprosy vaccination

Multidrug therapy (MDT) of leprosy was implemented by the WHO in 1981 to prevent and treat infections with drug-resistant strains as well as shorten leprosy treatment [10]. For practical treatment purpose, the WHO has established a simplified classification of leprosy patients. PB cases, showing ≤ 5 skin lesions, are treated for 6 months with a combination of rifampicin and dapsone, while MB cases, harboring > 5 skin lesions, are treated for a minimum of 12 months with a combination of rifampicin, dapsone, and clofazimine. MDT is highly efficient and free of charge for all patients [10]. Several clinical trials using the same drugs, but with shortened treatments, or with different therapeutic schemes, have been tested, but the efficacy was never higher than that of the current MDT regimen [10]. In parallel, cases of failure after 12 to 24 months of treatment are increasingly reported in Brazil [121]. Drug resistance, incorrect treatment, or host resistance, among others, might be causes of treatment failure. However, one of the main concerns of dermatologists is the use of only one bactericidal drug (rifampicin) in the main leprosy regimen.

There is currently no effective vaccine for leprosy. The gap in our understanding of the full pathogenesis of the bacteria and the host response in different stages of infection, including subclinical infections, is a detriment to any prospects of efficient vaccine development [4]. Several studies reported different percentages of protection conferred by the BCG vaccine, ranging from 30 to 50% [122]. Another approach was to combine the BCG vaccine with heat-inactivated *M. leprae* bacteria; however, no significant benefit was observed compared with BCG alone. Also, the mass implementation of this vaccine would have been challenging since *M. leprae* bacteria can be obtained only from infected humans or armadillo tissues [122, 123]. Analyzing the *M. leprae* genome revealed several vaccine candidate antigens. Several of these were tested as recombinant proteins with the primary objective to choose an antigen or a combination of antigens that induce an IFN-γ response against the bacteria [124, 125]. One promising vaccine candidate is LepVax, containing a 89-kD fusion protein called LEP-F1, consisting of the *M. leprae* proteins ML2028, ML2055, ML2380, and ML2531 formulated with a TLR4L adjuvant (Glucopyranosyl Lipid), stimulating the T_H_1 cytokine immune response [126]. Unlike BCG vaccination, which appears to trigger a reactional process causing neurologic damage, LepVax appears to be safe and may be able to induce a protective immune response in subclinically infected individuals without inducing inflammatory exacerbations.

#### Diagnosis of leprosy

Diagnosis of leprosy at early disease stages is essential to prevent disabilities. The gold standard is based on three cardinal signs: skin lesions, hypopigmented or reddish patches, with definite loss of sensation; enlargement of peripheral nerves associated or not with a weakness of the muscles supplied by that nerve; and the presence of AFBs in a slit-skin smear (SSS) [8]. Generally, the occurrence of skin lesions associated with sensory loss is a robust marker of leprosy. However, taken separately, the wide range of clinical symptoms might mislead patients and clinicians [4]. This is especially true when few or no lesions are visible in the case of PB patients, PNL, or at early stages of the disease. Several laboratory tests have been developed in the past 50 years with the aim to diagnose all leprosy forms and asymptomatic individuals.

The amount of AFBs in slit-skin smear or active lesions determines the bacterial load, also named bacillary or bacteriological index (BI). ZN staining is commonly used in peripheral centers to measure the BI. This low-cost method is specific, but lack of sensitivity, especially for samples with low BI and a BI of 0, is not a strong indication for the absence of disease. PCR-based tests targeting the repetitive element RLEP of *M. leprae* are currently the most sensitive methods to identify *M. leprae* in leprosy lesions especially for paucibacillary lesions [127]. However, similar to the BU field, these tests require good laboratory infrastructure, well-trained laboratory personnel, and strict adherence to quality control measures. That is why, in parallel to the improvement of molecular detection of the pathogen, there have been extensive research and development efforts for the design of simple, fast, and non-invasive diagnostic tests based on the immunological responses of the patients.

With the development of the specific di- and trisaccharide synthetic glycoconjugate mimetics of PGL-I called ND-O-BSA or ND-O-HSA, respectively, various assays and RDTs were developed to assess the anti-PGL-I antibody titers in leprosy patient sera [128]. Anti-PGL-I IgM antibodies are found in high quantity in the sera of MB patients (> 95% positivity) due to the increased humoral response, while they are often absent in PB patients (20–40% positivity) [129]. The RDT was available commercially as a lateral flow test, but the production was stopped before 2010 due to low demand and poor take-up by leprosy control programs [128]. Although studies have shown that there is a 6–8-fold higher risk of progressing to disease if positive [130], this test is not a good prognostic indicator of which of the infected individuals will progress to disease, as there is an overall resistance toward developing leprosy. Currently, assays to detect anti-PGL-I IgM titers are utilized mainly in high endemicity countries in the classification of patients to assist in assignment for the correct length of treatment, case monitoring and follow-up of household contacts at risk of developing disease, and the identification of individuals who are experiencing a relapse of their disease. To increase the sensitivity of detection in PB patients, a search for protein antigens that might be more suitable targets for a highly sensitive RDT was also initiated [131, 132]. The test sensitivity was increased in some studies, but the overall benefit compared with the detection of anti-PGL-I IgM was not significant [132].

In conjunction with investigations of the serological responses, various groups investigated also the possibility of developing a specific skin test or T cell cytokine release assay that could identify *M. leprae–*infected individuals, similar to the PPD delayed-type sensitivity skin test or the more sensitive and specific peptide-based Interferon Gamma Release Assay (IGRA) for *M. tuberculosis* infections [133]. With the completion of the annotation of whole-genome sequences of *M. leprae* and *M. tuberculosis*, comparative genomics allowed for the identification of low homology or unique *M. leprae* proteins that might be promising target antigens for the measurement of T cell responses [134]. Overall, over 200 recombinant proteins and peptides from *M. leprae* have been investigated as potential targets for serological and T cell–based diagnostic tests for *M. leprae* infection. Along those lines, two unique *M. leprae* proteins, ML2478 and ML0840, were shown to induce high IFN-γ responses in endemic control individuals from a high endemic country (Bangladesh) that were completely absent in control subjects from a non-endemic country (South Korea) [134, 135]. Recently, field-friendly lateral flow assays have been developed that can simultaneously detect both humoral anti-PGL-I IgM responses and cell-mediated responses (IP-10, CCL4, and C-reactive protein [CRP]) to the recombinant ML2478/ML0840 proteins and a whole-cell sonicate. The selection of the markers was driven by the aim to distinguish between different clinical leprosy types. Combined detection of all of these biomarkers significantly improved the diagnostic potential, particularly for PB leprosy in three endemic countries [135]. Simultaneous detection of anti-PGL-I IgM and cell-mediated biomarkers IP-10 and CRP was most recently tested using fingerstick blood to quantify humoral and cellular biomarkers indicative of *M. leprae* infection, allowing for a less invasive approach for the diagnosis of leprosy [135]. Longitudinal studies and additional validation of the newly developed RDTs are currently ongoing in different endemic and non-endemic countries.

### Conclusion: Intracellular *vs* necrotic hideout—a comparison of lifestyles

The current strategy to fight the neglected tropical skin diseases BU and leprosy relies on active case finding and antibiotic treatment, which in early stages of the diseases is curative and can prevent the disabling and stigmatizing long-term effects of the diseases. Both diseases have certain features in common but differ also profoundly in pathogenesis and transmission (Table 1). *M. ulcerans* and *M. leprae* both cause various types of skin lesions that can be relatively unspecific. This complicates the diagnosis of the two diseases, as no sufficiently sensitive and specific diagnostic point of care tests are available. If untreated, early lesions may evolve to serious dermatological and neurologic (leprosy) manifestations. The imminent threat of antibiotic resistance calls for new therapeutic options for both diseases.

**Table 1:** Comparison of BU and leprosy: common features and profound differences

Aspects	Buruli ulcer	Leprosy
Causative agent	*Mycobacterium ulcerans* (first isolated in 1948)	*Mycobacterium leprae* (first isolated in 1873); *Mycobacterium lepromatosis* (first isolated in 2008)
Clinical manifestations	Skin lesions	
	Pre-ulcerative nodules, papules, plaques, and edema; in advanced stages chronic, necrotizing ulcers affecting skin, subcutaneous tissue, and sometimes bones (osteomyelitis)	Papules, macules, nodules, plaques, hypochromic or not, scattered or disseminated; in specific forms: necrotizing ulcers, nerve lesions: thickness, tenderness, loss of sensation, hypo or total anesthesia, in advanced stages: deformities of the hands and feet, impairment of the eyes, soft tissues, and bones, endocrine dysfunction (sterility, osteoporosis, hypothyroidism)
WHO categories	Category I: single lesion < 5 cm in diameter. Category II: single lesion ≥ 5 to 15 cm in diameter, plaque, and edematous forms. Category III: single lesion > 15 cm in diameter, multiple lesions, lesion at critical sites, osteomyelitis	Paucibacillary: 5 or less skin lesions Multibacillary: more than 5 skin lesions
Diagnosis	Clinical signs and IS*2404* PCR in reference laboratories as gold standard for laboratory diagnosis; microscopy has limited sensitivity	Clinical signs and identification of bacteria in lesions; positive RLEP PCR in difficult to diagnose single lesions or pure neural leprosy cases
	Sensitive and specific point of care diagnostic tests needed	
Immunodiagnosis	No specific test available. In a large proportion of healthy individuals living in BU endemic areas serological responses and response to whole-cell lysate of *M. ulcerans*, most noticeable in people with healed lesions	None specific to all leprosy forms. Anti-PGL-I serological response is specific for multibacillary cases
Treatment	Combination therapy with rifampicin and clarithromycin for 8 weeks	Multibacillary: rifampicin, dapsone, and clofazimine, 12 months. Paucibacillary: rifampicin and dapsone, 6 months
	Shorter regimens with less severe side effects needed	
Genome	5.6 Mb, *~* 4160 CDS, 771 pseudogenes; 213 copies of IS*2404*, and 91 copies of IS*2606* (in strain Agy99); compared to *M. marinum* M (6.6 Mb), genome decay including loss of genes associated with intracellular lifestyle in other mycobacteria; acquisition of virulence plasmid (pMUM; 174 kb)	3.2 Mb, ~ 1600 CDS, 1100 pseudogenes; up to 37 copies of the specific repetitive element RLEP in *M. leprae*, absent in *M. lepromatosis*
	Genome reduction combined with loss of function mutations; niche-adapted pathogens	
Virulence factors	Cytotoxic and immunosuppressive effects of mycolactone; immune evasion facilitated by loss of immunodominant antigens	Unknown; probably ESX-1 system and PGL-I for neuropathy
Burden	More than 30,000 cases reported to WHO in the past 10 years; 2713 new cases in 2018; most likely substantial underreporting	> 200,000 new cases/year reported for the last 10 years; 208,613 new cases in 2018
Geographical distribution	Highly focal occurrence; most cases caused by classical lineage in West and Central Africa, Australia, PNG; sporadic cases by ancestral lineage in the Americas and Asia. Strong association of BU with stagnant water bodies	*M. leprae*: worldwide with 85% of new cases in India, Brazil, and Indonesia. *M. lepromatosis*: Mexico and the Caribbean region
Prevention	No vaccine candidate ready for clinical testing. Limited knowledge of transmission pathways and preventable risks	Vaccine in phase 1 clinical trial
Mode(s) of transmission	Unknown	
	Entry of mycobacteria into hosts presumably via penetration of skin through injuries and/or insect vectors; low probability of person-to-person transmission	Entry of mycobacteria into hosts presumably via the nose or skin; possible transmission via nasal droplets. Probable person-to-person transmission; zoonotic spread through contact with armadillos in the Southern United States and Brazil
Reservoir(s)	Unknown; presumed involvement of environmental and animal reservoirs; possums identified as animal reservoir in Australia	Mainly human; animal reservoirs: armadillos (USA), red squirrels (Europe), non-human primates (Africa, Philippines)
Incubation period	Can only be estimated due to limited knowledge on mode(s) of transmission. Mean incubation period of 4.5 months in Australia, may be shorter in Africa	3 to 20 years
Compartment	Extracellular in advanced lesions; early intracellular phase suspected	Obligate intracellular
Protective immunity	Mechanisms unknown; probably important role for cell-mediated immunity (against *M. ulcerans* in early disease stages)	
Effect of HIV infection	HIV infection increases both risk and severity of disease	No increase in risk but in severity due to the immune reconstitution inflammatory syndrome (IRIS) after initiation of HIV treatment
Immunological complications	Massive infiltration of lesions during antibiotic treatment; immune reconstitution inflammatory syndrome (IRIS)*-*like effects may in some patients cause delay in wound healing (“paradoxical reactions”)	Leprosy reactions (30–50% of cases) are characterized by an increased cellular response (reversal reaction). Mechanisms of ENL and Lucio’s phenomenon are unknown
Age spectrum and gender distribution	In Africa bimodal age distribution; BU mainly affects children between 4 and 15 years and the elderly population; overall balanced male:female ratio	All age groups affected; children are mostly diagnosed with mild forms such as indeterminate or tuberculoid leprosy
Host genetic factors	SNPs in the inducible nitric oxide synthase gene *iNOS* and in the interferon gamma gene *IFNG*, the natural resistance-associated macrophage protein gene *SLC11A1* (*NRAMP1*), and the autophagy-related E3 ubiquitin protein ligase gene *PARK2* have been linked to susceptibility to BU	SNPs in genes from the innate immune recognition, type I IFN, autophagy, lipid and energy metabolism, and the regulatory regions of PARK2 have been linked to susceptibility to leprosy
Spontaneous healing; exposure and disease	Anecdotal descriptions of spontaneous healing; sero-epidemiological studies indicate that only a small proportion of exposed individuals develop disease	
Long-term consequences	Catastrophic household expenditure for treatment; social isolation of patients during treatment and thereafter (stigma); permanent disabilities	

*M. ulcerans* and *M. leprae* patient isolates are characterized by limited genetic diversity. Both pathogens have gone through massive gene decay (loss of gene function through pseudogenization and genome reduction) in the course of their evolution, probably linked to the adaptation to new lifestyles and more stable niche environments [2, 12, 136]. *M. ulcerans* has evolved from a common progenitor with *M. marinum*, a process first and foremost enhanced by the acquisition of the virulence plasmid pMUM. It seems likely that the ability of *M. ulcerans* to produce mycolactone was a first step in the emergence of a highly clonal new species (MPM, which are now all designated *M. ulcerans*) with increased virulence compared with *M. marinum*, which only occasionally causes granulomatous skin infections in humans [137]. Additional changes in the chromosome, such as those leading to the loss of expression of the highly immunogenic proteins ESAT-6 and CFP-10 in *M. ulcerans*, also appear to contribute to the increase in virulence by reducing immunogenicity of the bacteria. On the other hand, this gene decay most likely also limits the ability of this largely extracellular pathogen to have an intracellular lifestyle. In contrast, the obligate intracellular leprosy bacilli have a functional ESX-1 virulence system, and there is no report of plasmid acquisition [136]. A key feature of the *M. leprae* genome is the even larger extent of gene deletions and of pseudogenization (pseudogenes occupy about 40% of the *M. leprae* chromosome). The resulting loss of gene function accounts for the failure to culture *M. leprae* in vitro, and loss of gene function mutations are most likely also responsible for the exceptionally slow growth rate of both *M. leprae* and *M. ulcerans*. Genomic data revealed that *M. leprae* strains have not drastically evolved in the past 4000 years, and the only breakthrough suggesting the existence of an *M. leprae* complex is the discovery of *M. lepromatosis* in Mexico [82, 138]. Both pathogens are able to cause leprosy-like lesions, but they genetically diverged 14 million years ago [82].

Leprosy is one of the oldest diseases known to mankind, having plagued humans for thousands of years. Despite the decrease in the prevalence of leprosy to less than 1 case per 100,000 population since the year 2000, the incidence has plateaued at 200,000 to 250,000 new reported cases per year globally over the past 10 years with the brunt of the burden falling on only a few countries in Asia, Africa, and Latin America. The first cases of BU were reported in the late nineteenth century, mainly in remote, rural areas of West and Central Africa, Papua New Guinea, and Australia. The disease may have been present for much longer periods of time in the remote endemic areas, and the apparent decline in the number of new BU cases reported in the past years is at least partly due to vast underreporting after a stark decline in financial support by key NGOs [11].

A more effective strategy to disease control would obviously be prevention, not least because BU and leprosy affect mainly poor rural populations with limited access to diagnostic and treatment facilities. Transmission is considered to be mainly interhuman in leprosy, whereas people in BU endemic areas are most likely primarily infected with *M. ulcerans* by contact with environmental reservoirs associated with aquatic ecosystems. Interhuman transmission of *M. ulcerans* seems to be very rare. Despite the successful worldwide administration of multidrug therapy for leprosy, transmission rates have remained stagnant. Therefore, the feasibility and acceptability of a single-dose preventative treatment with rifampicin given to direct contacts of newly diagnosed leprosy patients is currently being evaluated [139, 140]. The contribution of non-human reservoirs in the continuing transmission may be underestimated for leprosy, particularly in countries with unbroken high incidences. For *M. ulcerans*, no preventable risks are clearly identified, and if environmental reservoirs play a key role in transmission, early diagnosis and treatment of patients can only reduce incidence, if contamination of the environment via chronic BU wounds plays an important role in transmission. Effective preventive vaccination may not only protect individuals from developing BU or leprosy but may also interfere—at least in the case of leprosy—with transmission.

Mycobacterial pathogens have acquired vastly diverse and highly successful survival strategies in the host, bolstered by the impermeable nature of their cell wall providing resistance to many toxins and drugs. *M. tuberculosis*, one of the most prevalent human pathogens, is a facultative intracellular bacillus and has evolved to survive and replicate in host macrophages, to induce the formation of granulomas in which immune cells and bacteria colocalize, and to transit into a state of dormancy, which is extremely resistant to host defense [141]. Dormancy in tuberculosis gives rise to a large reservoir of latently infected individuals, in whom disease can reactivate at any time. Coinfection with HIV leads to a particularly high reactivation rate [142]. To date, dormancy in BU and leprosy has not been reported, but the incubation period of leprosy may be several years, constituting another reservoir for infection with unknown magnitude. *M. leprae* is an obligate intracellular mycobacterium, able to survive and multiply in phagocytic cells and to modulate the host immune system. In contrast, *M. ulcerans* replicates mainly extracellularly after a suspected early intracellular stage in the host, causing chronic infections by the mycolactone-mediated generation of necrotic infection foci, downregulation of host immune responses, and the killing of invading immune cells before they can reach the clusters of extracellular mycobacteria.

Depending on the polarization of T cell responses, a complex range of types of leprosy may occur. In tuberculoid leprosy patients, the growth of the mycobacteria is contained by T_H_1 cells that activate infected macrophages. As a consequence, few live bacteria are present, antibody production is limited, and local inflammation causes few skin lesions but peripheral nerve damage. Inhibition of the T_H_1 responses to *M. leprae* leads to the severe, disseminated lepromatous form of the disease, in which a predominant T_H_2 response is unable to control replication of the mycobacteria in macrophages, leading to severe damage to connective tissue and the peripheral nervous system, if untreated. Knowledge on cell-mediated protective immune responses to BU is limited. As cell-mediated immunity is likely to play an important role in a postulated early, intracellular stage of *M. ulcerans* infections, a scenario similar to leprosy may be envisaged, in which T_H_1 responses lead to protective immunity against BU, whereas T_H_2 responses may be detrimental. The polarization of T cell responses in BU and leprosy may be influenced by host factors, the route of infection, and/or the dose of the initial inoculum.

While HIV infection increases the risk and severity of BU, this is not the case for leprosy. But initiation of HIV treatment may for both diseases bring the danger of an immune reconstitution inflammatory syndrome. Massive leukocyte infiltrations are also observed in BU lesions after initiation of antimycobacterial chemotherapy, when mycolactone levels drop and infiltrating immune cells are no longer killed by the cytotoxin. Also, host genetic factors are likely to play an important role for the outcome of an exposure to *M. ulcerans* and *M. leprae*. Case-control studies have identified significant associations of susceptibility to BU and leprosy with polymorphisms that are relevant for cellular immunity. However, selection of candidate genes investigated so far was strongly biased toward host polymorphisms that have previously shown significant associations with susceptibility to intracellular mycobacteria, and genome-wide association studies may in future identify genes relevant for other immune effector functions.

Vaccine design for mycobacterial diseases is hampered by the distinct resistance of the bacilli to many immune defense mechanisms and a lack of knowledge on immune effector functions required for protective immunity. Contact to the mycobacterial pathogens does lead only in a minority of individuals to clinical disease, and spontaneous healing seems to occur in both BU and leprosy. Lessons may be learnt by identifying factors leading to this highly diverse individual outcome of exposure to the pathogens and by analyzing responses to BCG vaccination. Although the precise mechanisms of protection conferred by BCG are not clearly understood, studies have demonstrated that BCG vaccination induces cellular immune responses that can limit the bacterial burden at the site of infection [143]. BCG vaccination has however been shown to convey only limited protection from pulmonary tuberculosis [144] and leprosy [145], and only short-lived protection against BU [70]. On the contrary, BCG is effective at preventing childhood tuberculosis meningitis and disseminated tuberculosis [146] as well as BU-associated osteomyelitis [68, 69]. It is tempting to speculate that by reducing the bacterial burden through early cell-mediated immune responses elicited by BCG, severe forms of the infections may be prevented, whereas these responses are not rigorous enough to entirely contain the pathogenic effects of the mycobacterial pathogens. In this context, it has been postulated in recent years that BCG vaccination has the potential to induce a short-lived, non-specific, cross-protective memory-like response in innate immune cells, a phenomenon known as “trained immunity,” responsible for early clearance of the pathogens and disease prevention [147–149].

Understanding the exact mechanism of the immunopathology of BU and leprosy will help in developing new strategies for the design of effective vaccines and specific and highly sensitive RDTs. Mycolactone is the most promising target for future vaccine design for BU and also for the development of a point of care diagnostic test. Whereas the complexity of clinical leprosy forms, immunological responses, and variations in bacillary load in lesions have hindered the development of a universal diagnostic test for leprosy, the subunit vaccine candidate LepVax has recently entered clinical testing [126]. In comparison, the tuberculosis vaccine pipeline is much broader, and in view of similarities in the antigenic makeup of *M. tuberculosis*, *M. leprae*, and *M. ulcerans*, there is hope that a newly developed effective TB vaccine may be cross-protective against leprosy and BU.

## Footnotes

1 As treatment with streptomycin has been shown to cause permanent ototoxicity and transient nephrotoxicity in BU patients, the WHO Technical Advisory Group on Buruli ulcer recommended in 2017 replacing streptomycin with oral clarithromycin for the treatment of BU in Africa.

2 It is currently not possible to distinguish between *M. leprae* and *M. lepromatosis* infection without molecular tools. Less than 20 cases of *M. lepromatosis* infection have been reported worldwide mostly in Mexico and the Caribbean region with a few sporadic cases in Asia, Europe, and North America. There have been not enough investigations regarding the differences in clinical outcomes and immunological responses between both pathogens. Therefore, the information reported in this review concerns *M. leprae* infections.
